# Importation and Containment of Ebola Virus Disease — Senegal, August–September 2014

**Published:** 2014-10-03

**Authors:** Kelsey Mirkovic, Julie Thwing, Papa Amadou Diack

**Affiliations:** 1 Epidemic Intelligence Service, CDC; 2 Division of Nutrition, Physical Activity, and Obesity, National Center for Chronic Disease Prevention and Health Promotion, CDC; 3 Division of Parasitic Diseases and Malaria, Center for Global Health, CDC; 4 Senegal Ministry of Health and Social Welfare; 5 Global Immunization Division, Centers for Global Health, CDC; 6 Division of High-Consequence Pathogens and Pathology, National Center for Emerging and Zoonotic Infectious Diseases, CDC; 7 World Health Organization, Regional Office for Europe; 8 World Health Organization, Regional Office for Africa

On August 29, 2014, Senegal confirmed its first case of Ebola virus disease (Ebola) in a Guinean man, aged 21 years, who had traveled from Guinea to Dakar, Senegal, in mid-August to visit family. Senegalese medical and public health personnel were alerted about this patient after public health staff in Guinea contacted his family in Senegal on August 27. The patient had been admitted to a referral hospital in Senegal on August 26. He was promptly isolated, and a blood sample was sent for laboratory confirmation; Ebola was confirmed by reverse transcriptase–polymerase chain reaction at Institut Pasteur Dakar on August 29. The patient’s mother and sister had been admitted to an Ebola treatment unit in Guinea on August 26, where they had named the patient as a contact and reported his recent travel to Senegal. Ebola was likely transmitted to the family from the brother of the patient, who had traveled by land from Sierra Leone to Guinea in early August seeking treatment from a traditional healer. The brother died in Guinea on August 10; family members, including the patient, participated in preparing the body for burial.

Although details about the timing of disease progression obtained by interviewing the patient and the family were inconsistent, the best information suggests that the patient arrived in Senegal by seven-person taxi, on or around August 14 and began experiencing fever, diarrhea, and vomiting on August 16. He initially sought care at a neighborhood health post on or around August 18, where he continued follow-up as an outpatient until August 25. During this time, he received intravenous fluids and other symptomatic treatment. On August 26, he was admitted to the University Hospital Fann, a tertiary care hospital in Dakar. The patient did not disclose a history of travel or contact with any Ebola patients.

Before this occurrence of the first confirmed case of Ebola in Senegal, the Senegal Ministry of Health had been preparing for the possible introduction of an imported case. Training of health care staff had been conducted on Ebola and infection control, laboratory testing, case investigation, and contact tracing, with an oversight committee organized for response. A total of 67 contacts of the patient were initially identified: 34 residents of the home where the patient stayed and 33 health care workers. Because of uncertainty regarding the timeline of the patient’s illness, all contacts were subjected to a 21-day monitoring period beginning on August 29. Contacts were requested to submit to in-home voluntary quarantine and be seen twice daily by Red Cross volunteers mobilized as contact monitors. Symptoms and temperatures were recorded twice daily. Food was provided for the household contacts.

On the first day of monitoring, 51% of contacts were seen; this increased to over 90% by day 5. Household member contacts complied with monitoring throughout the quarantine period, but some health care worker contacts resisted monitoring by Red Cross volunteers. Discussion with health care worker contacts suggested that some of them opposed in-person temperature monitoring by Red Cross volunteers. Alternative solutions were sought, and monitoring was reassigned to University Hospital Fann’s personnel for resistant health care worker contacts, which resulted in increased compliance. On day 13 of follow-up, an additional seven exposed workers from University Hospital Fann self-identified during training on infection control, and they underwent voluntary restriction of movement and temperature monitoring through the 21st day after exposure. During monitoring, four contacts developed transient symptoms suggestive of Ebola, but Ebola was ruled out by laboratory testing. All 67 contacts completed the 21-day follow-up on September 18 with no further confirmed Ebola cases. The patient recovered and was released from isolation on September 19. Before the confirmation of this case and during the contact follow-up, numerous unrelated suspected cases were identified, tested, and found to be negative.

Prompt notification of health personnel in Senegal about the case by health personnel in Guinea, and early preparations by the Ministry of Health and partners in Senegal for anticipated imported cases of Ebola, resulted in a rapid containment response. Prompt notification through an interagency collaboration in Guinea was crucial in this case because the patient did not report recent travel or contact with an Ebola patient. An incident command structure is being adopted by the Senegal Ministry of Health to prepare for any additional cases, and surveillance systems continue to be strengthened.

The current Ebola epidemic in West Africa is unprecedented. As of September 23, 2014, the World Health Organization reported 6,574 cases with 3,091 deaths (1). Currently, the epidemic is primarily affecting Guinea, Liberia, and Sierra Leone; however, active trade and ease of travel in West Africa leave neighboring countries at risk for Ebola importation. Nigeria reported its first imported case of Ebola in July (2), and Senegal was the fifth West African country to be affected.

Ebola is a serious threat to West Africa, especially countries that border the heavily affected areas. Although there are systems in place for health screening at international airports in Ebola-affected countries, land border crossings do not provide the same limited points of departure and entry and associated opportunities for health screening. A framework for rapid Ebola identification and containment is needed urgently in all West African countries, including a strong system for cross-border communication. Difficulties related to coordination and implementation of policies and procedures are likely to occur, necessitating thorough planning and rapid troubleshooting. To prepare for a possible Ebola importation, it is important for bordering countries to have an active Ebola health care surveillance system and establish an incident command structure that is ready to be activated if necessary. It is important for neighboring countries to anticipate imported cases and define success as containment rather than exclusion of imported Ebola cases.
